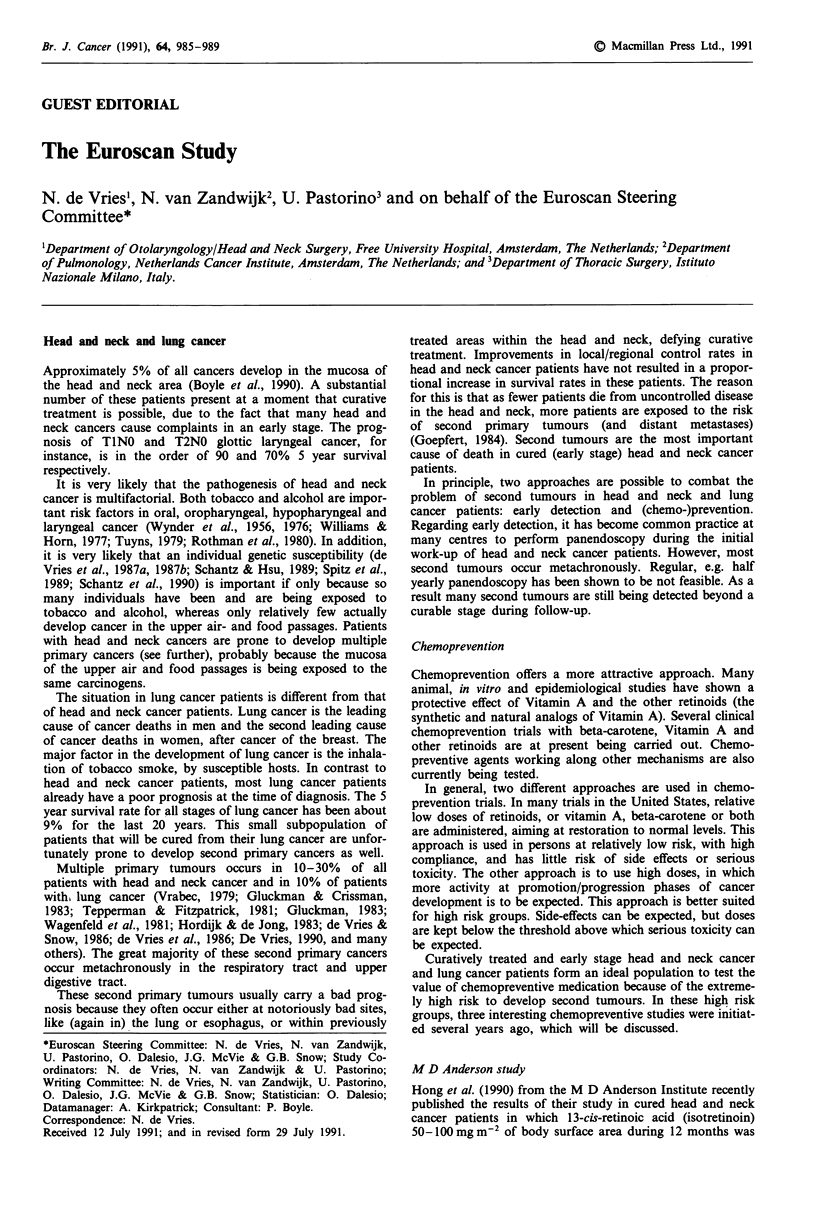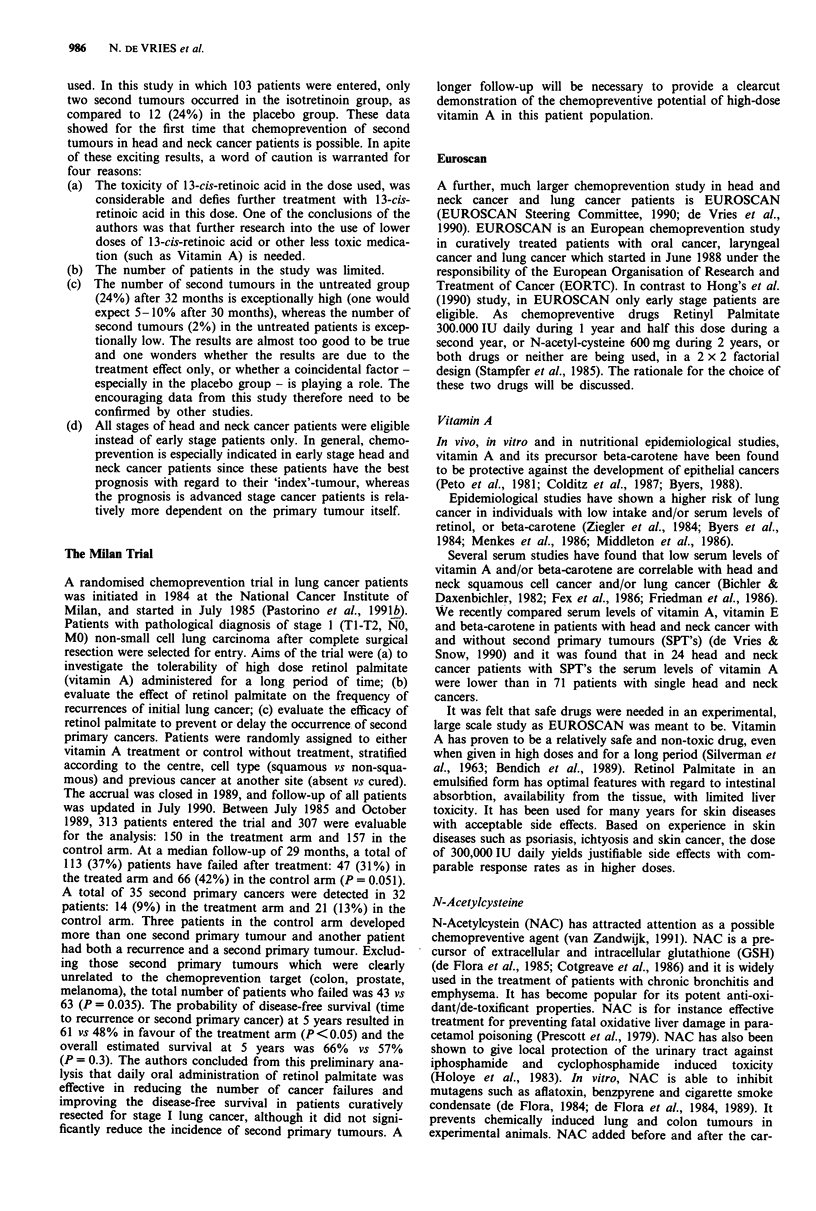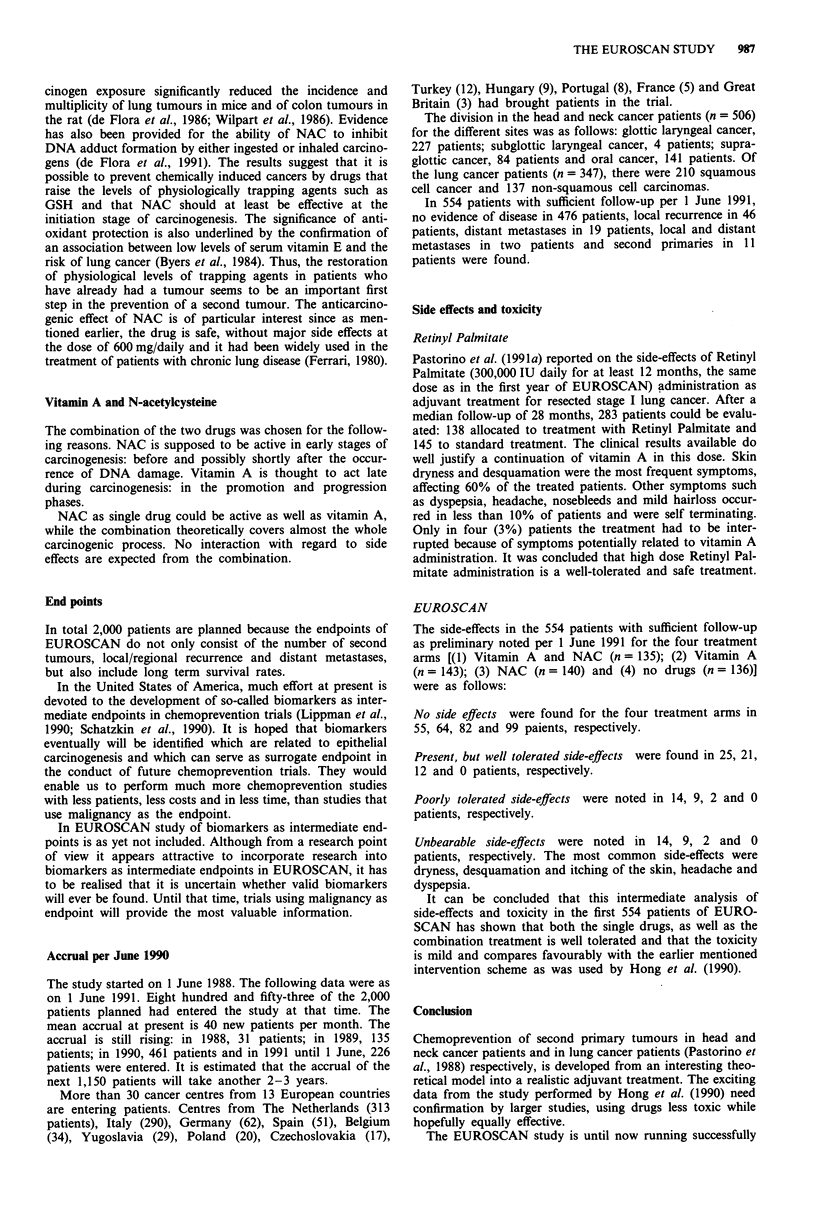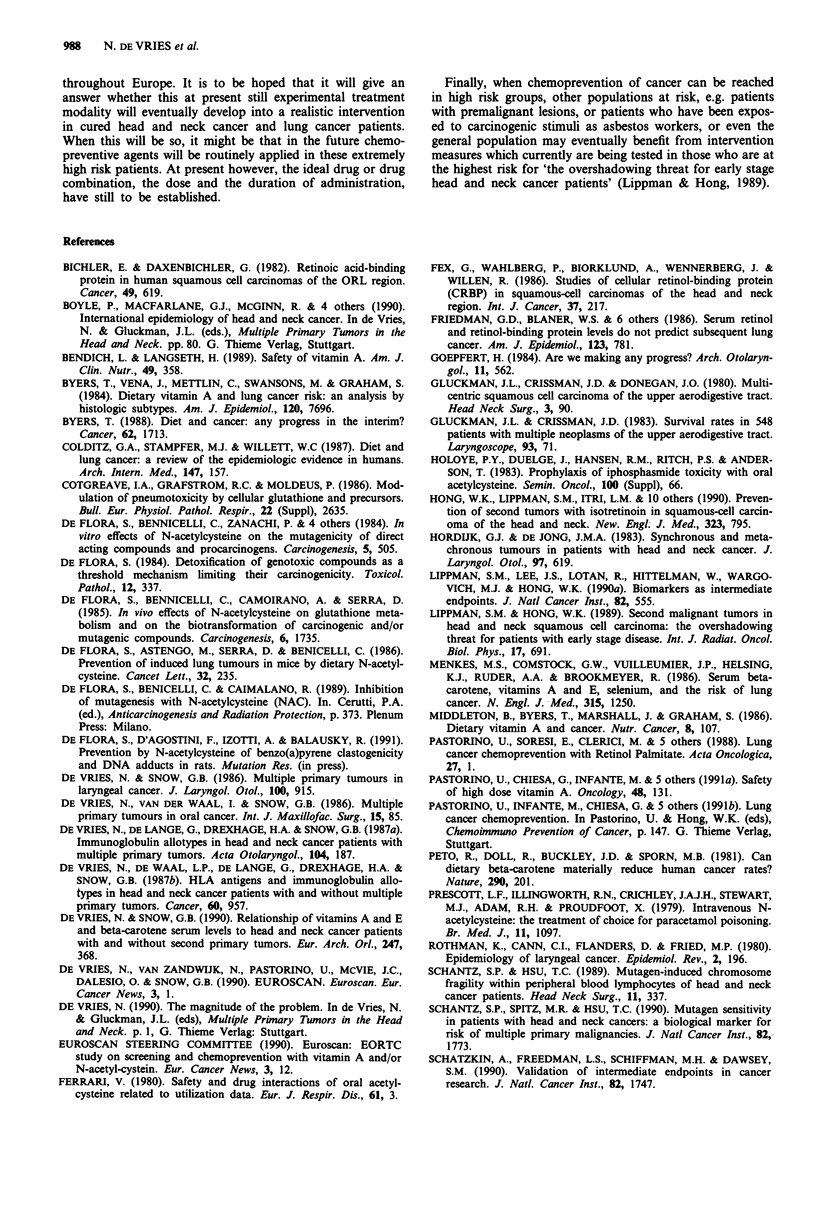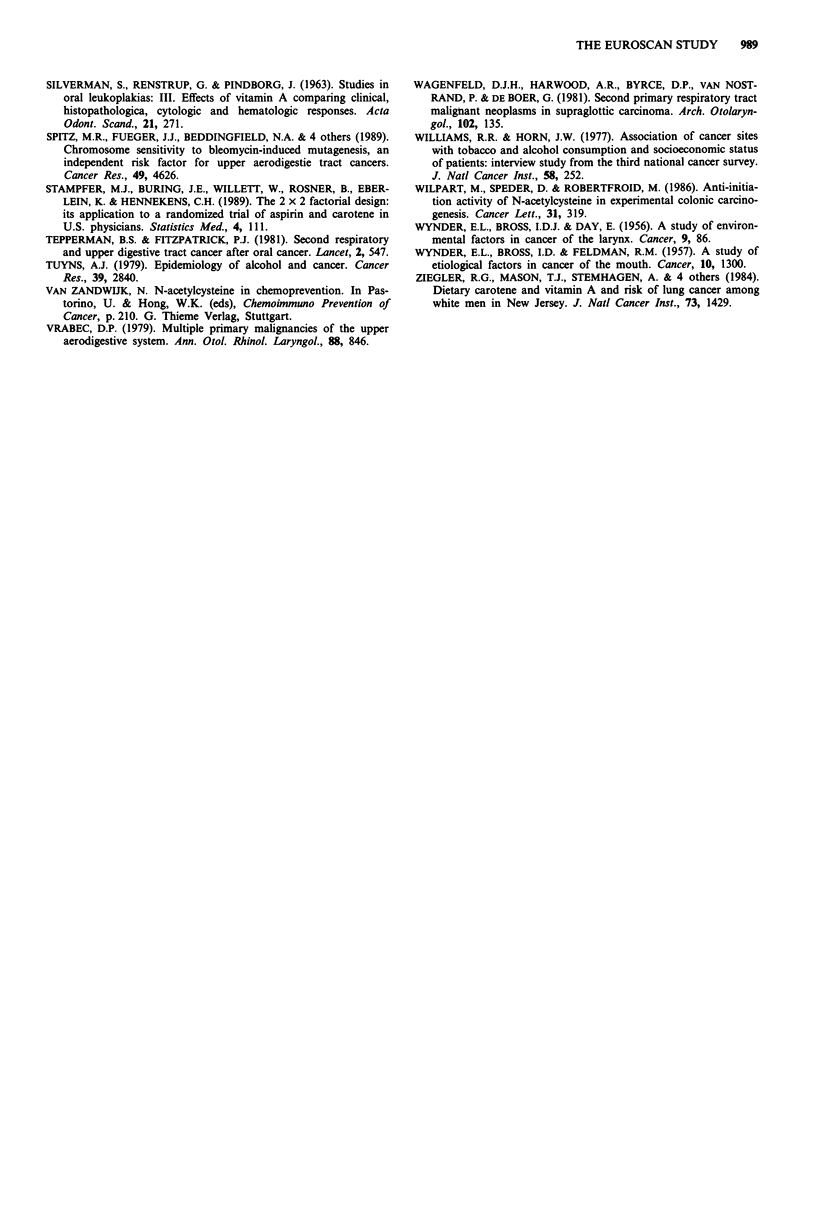# The EUROSCAN Study. EUROSCAN Steering Committee.

**DOI:** 10.1038/bjc.1991.451

**Published:** 1991-12

**Authors:** N. de Vries, N. van Zandwijk, U. Pastorino


Br. J. Cancer (1991), 64, 985–989 © Macmillan Press Ltd., 1991

GUEST EDITORIAL

### The Euroscan Study

N. de Vries[1], N. van Zandwijk[2], U. Pastorino[3] and on behalf of the Euroscan Steering Committee*

[1]*Department of Otolaryngology/Head and Neck Surgery, Free University Hospital, Amsterdam, The Netherlands;* [2]*Department of Pulmonology, Netherlands Cancer Institute, Amsterdam, The Netherlands; and* [3]*Department of Thoracic Surgery, Istituto Nazionale Milano, Italy.*

### Head and neck and lung cancer

Approximately 5% of all cancers develop in the mucosa of the head and neck area (Boyle *et al.*, 1990). A substantial number of these patients present at a moment that curative treatment is possible, due to the fact that many head and neck cancers cause complaints in an early stage. The prognosis of T1N0 and T2N0 glottic laryngeal cancer, for instance, is in the order of 90 and 70% 5 year survival respectively.

It is very likely that the pathogenesis of head and neck cancer is multifactorial. Both tobacco and alcohol are important risk factors in oral, oropharyngeal, hypopharyngeal and laryngeal cancer (Wynder *et al.*, 1956, 1976; Williams & Horn, 1977; Tuyns, 1979; Rothman *et al.*, 1980). In addition, it is very likely that an individual genetic susceptibility (de Vries *et al.*, 1987*a*, 1987*b*; Schantz & Hsu, 1989; Spitz *et al.*, 1989; Schantz *et al.*, 1990) is important if only because so many individuals have been and are being exposed to tobacco and alcohol, whereas only relatively few actually develop cancer in the upper air- and food passages. Patients with head and neck cancers are prone to develop multiple primary cancers (see further), probably because the mucosa of the upper air and food passages is being exposed to the same carcinogens.

The situation in lung cancer patients is different from that of head and neck cancer patients. Lung cancer is the leading cause of cancer deaths in men and the second leading cause of cancer deaths in women, after cancer of the breast. The major factor in the development of lung cancer is the inhalation of tobacco smoke, by susceptible hosts. In contrast to head and neck cancer patients, most lung cancer patients already have a poor prognosis at the time of diagnosis. The 5 year survival rate for all stages of lung cancer has been about 9% for the last 20 years. This small subpopulation of patients that will be cured from their lung cancer are unfortunately prone to develop second primary cancers as well.

Multiple primary tumours occurs in 10–30% of all patients with head and neck cancer and in 10% of patients with lung cancer (Vrabec, 1979; Gluckman & Crissman, 1983; Tepperman & Fitzpatrick, 1981; Gluckman, 1983; Wagenfeld *et al.*, 1981; Hordijk & de Jong, 1983; de Vries & Snow, 1986; de Vries *et al.*, 1986; De Vries, 1990, and many others). The great majority of these second primary cancers occur metachronously in the respiratory tract and upper digestive tract.

These second primary tumours usually carry a bad prognosis because they often occur either at notoriously bad sites, like (again in) the lung or esophagus, or within previously treated areas within the head and neck, defying curative treatment. Improvements in local/regional control rates in head and neck cancer patients have not resulted in a proportional increase in survival rates in these patients. The reason for this is that as fewer patients die from uncontrolled disease in the head and neck, more patients are exposed to the risk of second primary tumours (and distant metastases) (Goepfert, 1984). Second tumours are the most important cause of death in cured (early stage) head and neck cancer patients.

In principle, two approaches are possible to combat the problem of second tumours in head and neck and lung cancer patients: early detection and (chemo-)prevention. Regarding early detection, it has become common practice at many centres to perform panendoscopy during the initial work-up of head and neck cancer patients. However, most second tumours occur metachronously. Regular, e.g. half yearly panendoscopy has been shown to be not feasible. As a result many second tumours are still being detected beyond a curable stage during follow-up.

### *Chemoprevention*

Chemoprevention offers a more attractive approach. Many animal, *in vitro* and epidemiological studies have shown a protective effect of Vitamin A and the other retinoids (the synthetic and natural analogs of Vitamin A). Several clinical chemoprevention trials with beta-carotene, Vitamin A and other retinoids are at present being carried out. Chemopreventive agents working along other mechanisms are also currently being tested.

In general, two different approaches are used in chemoprevention trials. In many trials in the United States, relative low doses of retinoids, or vitamin A, beta-carotene or both are administered, aiming at restoration to normal levels. This approach is used in persons at relatively low risk, with high compliance, and has little risk of side effects or serious toxicity. The other approach is to use high doses, in which more activity at promotion/progression phases of cancer development is to be expected. This approach is better suited for high risk groups. Side-effects can be expected, but doses are kept below the threshold above which serious toxicity can be expected.

Curatively treated and early stage head and neck cancer and lung cancer patients form an ideal population to test the value of chemopreventive medication because of the extremely high risk to develop second tumours. In these high risk groups, three interesting chemopreventive studies were initiated several years ago, which will be discussed.

### *M D Anderson study*

Hong *et al.* (1990) from the M D Anderson Institute recently published the results of their study in cured head and neck cancer patients in which 13-*cis*-retinoic acid (isotretinoin) 50–100 mg $m^{-2}$ of body surface area during 12 months was

*Euroscan Steering Committee: N. de Vries, N. van Zandwijk, U. Pastorino, O. Dalesio, J.G. McVie & G.B. Snow; Study Coordinators: N. de Vries, N. van Zandwijk & U. Pastorino; Writing Committee: N. de Vries, N. van Zandwijk, U. Pastorino, O. Dalesio, J.G. McVie & G.B. Snow; Statistician: O. Dalesio; Datamanager: A. Kirkpatrick; Consultant: P. Boyle.
Correspondence: N. de Vries.
Received 12 July 1991; and in revised form 29 July 1991.

used. In this study in which 103 patients were entered, only two second tumours occurred in the isotretinoin group, as compared to 12 (24%) in the placebo group. These data showed for the first time that chemoprevention of second tumours in head and neck cancer patients is possible. In apite of these exciting results, a word of caution is warranted for four reasons:

(a) The toxicity of 13-*cis*-retinoic acid in the dose used, was considerable and defies further treatment with 13-*cis*-retinoic acid in this dose. One of the conclusions of the authors was that further research into the use of lower doses of 13-*cis*-retinoic acid or other less toxic medication (such as Vitamin A) is needed.
(b) The number of patients in the study was limited.
(c) The number of second tumours in the untreated group (24%) after 32 months is exceptionally high (one would expect 5–10% after 30 months), whereas the number of second tumours (2%) in the untreated patients is exceptionally low. The results are almost too good to be true and one wonders whether the results are due to the treatment effect only, or whether a coincidental factor – especially in the placebo group – is playing a role. The encouraging data from this study therefore need to be confirmed by other studies.
(d) All stages of head and neck cancer patients were eligible instead of early stage patients only. In general, chemoprevention is especially indicated in early stage head and neck cancer patients since these patients have the best prognosis with regard to their 'index'-tumour, whereas the prognosis is advanced stage cancer patients is relatively more dependent on the primary tumour itself.

**The Milan Trial**

A randomised chemoprevention trial in lung cancer patients was initiated in 1984 at the National Cancer Institute of Milan, and started in July 1985 (Pastorino *et al.*, 1991*b*). Patients with pathological diagnosis of stage 1 (T1-T2, N0, M0) non-small cell lung carcinoma after complete surgical resection were selected for entry. Aims of the trial were (a) to investigate the tolerability of high dose retinol palmitate (vitamin A) administered for a long period of time; (b) evaluate the effect of retinol palmitate on the frequency of recurrences of initial lung cancer; (c) evaluate the efficacy of retinol palmitate to prevent or delay the occurrence of second primary cancers. Patients were randomly assigned to either vitamin A treatment or control without treatment, stratified according to the centre, cell type (squamous *vs* non-squamous) and previous cancer at another site (absent *vs* cured). The accrual was closed in 1989, and follow-up of all patients was updated in July 1990. Between July 1985 and October 1989, 313 patients entered the trial and 307 were evaluable for the analysis: 150 in the treatment arm and 157 in the control arm. At a median follow-up of 29 months, a total of 113 (37%) patients have failed after treatment: 47 (31%) in the treated arm and 66 (42%) in the control arm ($P = 0.051$). A total of 35 second primary cancers were detected in 32 patients: 14 (9%) in the treatment arm and 21 (13%) in the control arm. Three patients in the control arm developed more than one second primary tumour and another patient had both a recurrence and a second primary tumour. Excluding those second primary tumours which were clearly unrelated to the chemoprevention target (colon, prostate, melanoma), the total number of patients who failed was 43 *vs* 63 ($P = 0.035$). The probability of disease-free survival (time to recurrence or second primary cancer) at 5 years resulted in 61 *vs* 48% in favour of the treatment arm ($P < 0.05$) and the overall estimated survival at 5 years was 66% *vs* 57% ($P = 0.3$). The authors concluded from this preliminary analysis that daily oral administration of retinol palmitate was effective in reducing the number of cancer failures and improving the disease-free survival in patients curatively resected for stage I lung cancer, although it did not significantly reduce the incidence of second primary tumours. A longer follow-up will be necessary to provide a clearcut demonstration of the chemopreventive potential of high-dose vitamin A in this patient population.

**Euroscan**

A further, much larger chemoprevention study in head and neck cancer and lung cancer patients is EUROSCAN (EUROSCAN Steering Committee, 1990; de Vries *et al.*, 1990). EUROSCAN is an European chemoprevention study in curatively treated patients with oral cancer, laryngeal cancer and lung cancer which started in June 1988 under the responsibility of the European Organisation of Research and Treatment of Cancer (EORTC). In contrast to Hong's *et al.* (1990) study, in EUROSCAN only early stage patients are eligible. As chemopreventive drugs Retinyl Palmitate 300.000 IU daily during 1 year and half this dose during a second year, or N-acetyl-cysteine 600 mg during 2 years, or both drugs or neither are being used, in a 2 × 2 factorial design (Stampfer *et al.*, 1985). The rationale for the choice of these two drugs will be discussed.

*Vitamin A*

*In vivo*, *in vitro* and in nutritional epidemiological studies, vitamin A and its precursor beta-carotene have been found to be protective against the development of epithelial cancers (Peto *et al.*, 1981; Colditz *et al.*, 1987; Byers, 1988).

Epidemiological studies have shown a higher risk of lung cancer in individuals with low intake and/or serum levels of retinol, or beta-carotene (Ziegler *et al.*, 1984; Byers *et al.*, 1984; Menkes *et al.*, 1986; Middleton *et al.*, 1986).

Several serum studies have found that low serum levels of vitamin A and/or beta-carotene are correlable with head and neck squamous cell cancer and/or lung cancer (Bichler & Daxenbichler, 1982; Fex *et al.*, 1986; Friedman *et al.*, 1986). We recently compared serum levels of vitamin A, vitamin E and beta-carotene in patients with head and neck cancer with and without second primary tumours (SPT's) (de Vries & Snow, 1990) and it was found that in 24 head and neck cancer patients with SPT's the serum levels of vitamin A were lower than in 71 patients with single head and neck cancers.

It was felt that safe drugs were needed in an experimental, large scale study as EUROSCAN was meant to be. Vitamin A has proven to be a relatively safe and non-toxic drug, even when given in high doses and for a long period (Silverman *et al.*, 1963; Bendich *et al.*, 1989). Retinol Palmitate in an emulsified form has optimal features with regard to intestinal absorbtion, availability from the tissue, with limited liver toxicity. It has been used for many years for skin diseases with acceptable side effects. Based on experience in skin diseases such as psoriasis, ichtyosis and skin cancer, the dose of 300,000 IU daily yields justifiable side effects with comparable response rates as in higher doses.

*N-Acetylcysteine*

N-Acetylcystein (NAC) has attracted attention as a possible chemopreventive agent (van Zandwijk, 1991). NAC is a precursor of extracellular and intracellular glutathione (GSH) (de Flora *et al.*, 1985; Cotgreave *et al.*, 1986) and it is widely used in the treatment of patients with chronic bronchitis and emphysema. It has become popular for its potent anti-oxidant/de-toxificant properties. NAC is for instance effective treatment for preventing fatal oxidative liver damage in paracetamol poisoning (Prescott *et al.*, 1979). NAC has also been shown to give local protection of the urinary tract against iphosphamide and cyclophosphamide induced toxicity (Holoye *et al.*, 1983). *In vitro*, NAC is able to inhibit mutagens such as aflatoxin, benzpyrene and cigarette smoke condensate (de Flora, 1984; de Flora *et al.*, 1984, 1989). It prevents chemically induced lung and colon tumours in experimental animals. NAC added before and after the carcinogen exposure significantly reduced the incidence and multiplicity of lung tumours in mice and of colon tumours in the rat (de Flora *et al*., 1986; Wilpart *et al*., 1986). Evidence has also been provided for the ability of NAC to inhibit DNA adduct formation by either ingested or inhaled carcinogens (de Flora *et al*., 1991). The results suggest that it is possible to prevent chemically induced cancers by drugs that raise the levels of physiologically trapping agents such as GSH and that NAC should at least be effective at the initiation stage of carcinogenesis. The significance of antioxidant protection is also underlined by the confirmation of an association between low levels of serum vitamin E and the risk of lung cancer (Byers *et al*., 1984). Thus, the restoration of physiological levels of trapping agents in patients who have already had a tumour seems to be an important first step in the prevention of a second tumour. The anticarcinogenic effect of NAC is of particular interest since as mentioned earlier, the drug is safe, without major side effects at the dose of 600 mg/daily and it had been widely used in the treatment of patients with chronic lung disease (Ferrari, 1980).

**Vitamin A and N-acetylcysteine**

The combination of the two drugs was chosen for the following reasons. NAC is supposed to be active in early stages of carcinogenesis: before and possibly shortly after the occurrence of DNA damage. Vitamin A is thought to act late during carcinogenesis: in the promotion and progression phases.

NAC as single drug could be active as well as vitamin A, while the combination theoretically covers almost the whole carcinogenic process. No interaction with regard to side effects are expected from the combination.

**End points**

In total 2,000 patients are planned because the endpoints of EUROSCAN do not only consist of the number of second tumours, local/regional recurrence and distant metastases, but also include long term survival rates.

In the United States of America, much effort at present is devoted to the development of so-called biomarkers as intermediate endpoints in chemoprevention trials (Lippman *et al*., 1990; Schatzkin *et al*., 1990). It is hoped that biomarkers eventually will be identified which are related to epithelial carcinogenesis and which can serve as surrogate endpoint in the conduct of future chemoprevention trials. They would enable us to perform much more chemoprevention studies with less patients, less costs and in less time, than studies that use malignancy as the endpoint.

In EUROSCAN study of biomarkers as intermediate endpoints is as yet not included. Although from a research point of view it appears attractive to incorporate research into biomarkers as intermediate endpoints in EUROSCAN, it has to be realised that it is uncertain whether valid biomarkers will ever be found. Until that time, trials using malignancy as endpoint will provide the most valuable information.

**Accrual per June 1990**

The study started on 1 June 1988. The following data were as on 1 June 1991. Eight hundred and fifty-three of the 2,000 patients planned had entered the study at that time. The mean accrual at present is 40 new patients per month. The accrual is still rising: in 1988, 31 patients; in 1989, 135 patients; in 1990, 461 patients and in 1991 until 1 June, 226 patients were entered. It is estimated that the accrual of the next 1,150 patients will take another 2–3 years.

More than 30 cancer centres from 13 European countries are entering patients. Centres from The Netherlands (313 patients), Italy (290), Germany (62), Spain (51), Belgium (34), Yugoslavia (29), Poland (20), Czechoslovakia (17), Turkey (12), Hungary (9), Portugal (8), France (5) and Great Britain (3) had brought patients in the trial.

The division in the head and neck cancer patients ($n = 506$) for the different sites was as follows: glottic laryngeal cancer, 227 patients; subglottic laryngeal cancer, 4 patients; supraglottic cancer, 84 patients and oral cancer, 141 patients. Of the lung cancer patients ($n = 347$), there were 210 squamous cell cancer and 137 non-squamous cell carcinomas.

In 554 patients with sufficient follow-up per 1 June 1991, no evidence of disease in 476 patients, local recurrence in 46 patients, distant metastases in 19 patients, local and distant metastases in two patients and second primaries in 11 patients were found.

**Side effects and toxicity**

*Retinyl Palmitate*

Pastorino *et al*. (1991*a*) reported on the side-effects of Retinyl Palmitate (300,000 IU daily for at least 12 months, the same dose as in the first year of EUROSCAN) administration as adjuvant treatment for resected stage I lung cancer. After a median follow-up of 28 months, 283 patients could be evaluated: 138 allocated to treatment with Retinyl Palmitate and 145 to standard treatment. The clinical results available do well justify a continuation of vitamin A in this dose. Skin dryness and desquamation were the most frequent symptoms, affecting 60% of the treated patients. Other symptoms such as dyspepsia, headache, nosebleeds and mild hairloss occurred in less than 10% of patients and were self terminating. Only in four (3%) patients the treatment had to be interrupted because of symptoms potentially related to vitamin A administration. It was concluded that high dose Retinyl Palmitate administration is a well-tolerated and safe treatment.

*EUROSCAN*

The side-effects in the 554 patients with sufficient follow-up as preliminary noted per 1 June 1991 for the four treatment arms [(1) Vitamin A and NAC ($n = 135$); (2) Vitamin A ($n = 143$); (3) NAC ($n = 140$) and (4) no drugs ($n = 136$)] were as follows:

*No side effects* were found for the four treatment arms in 55, 64, 82 and 99 paients, respectively.

*Present, but well tolerated side-effects* were found in 25, 21, 12 and 0 patients, respectively.

*Poorly tolerated side-effects* were noted in 14, 9, 2 and 0 patients, respectively.

*Unbearable side-effects* were noted in 14, 9, 2 and 0 patients, respectively. The most common side-effects were dryness, desquamation and itching of the skin, headache and dyspepsia.

It can be concluded that this intermediate analysis of side-effects and toxicity in the first 554 patients of EUROSCAN has shown that both the single drugs, as well as the combination treatment is well tolerated and that the toxicity is mild and compares favourably with the earlier mentioned intervention scheme as was used by Hong *et al*. (1990).

**Conclusion**

Chemoprevention of second primary tumours in head and neck cancer patients and in lung cancer patients (Pastorino *et al*., 1988) respectively, is developed from an interesting theoretical model into a realistic adjuvant treatment. The exciting data from the study performed by Hong *et al*. (1990) need confirmation by larger studies, using drugs less toxic while hopefully equally effective.

The EUROSCAN study is until now running successfully throughout Europe. It is to be hoped that it will give an answer whether this at present still experimental treatment modality will eventually develop into a realistic intervention in cured head and neck cancer and lung cancer patients. When this will be so, it might be that in the future chemopreventive agents will be routinely applied in these extremely high risk patients. At present however, the ideal drug or drug combination, the dose and the duration of administration, have still to be established.

Finally, when chemoprevention of cancer can be reached in high risk groups, other populations at risk, e.g. patients with premalignant lesions, or patients who have been exposed to carcinogenic stimuli as asbestos workers, or even the general population may eventually benefit from intervention measures which currently are being tested in those who are at the highest risk for 'the overshadowing threat for early stage head and neck cancer patients' (Lippman & Hong, 1989).

### References

BICHLER, E. & DAXENBICHLER, G. (1982). Retinoic acid-binding protein in human squamous cell carcinomas of the ORL region. *Cancer*, **49**, 619.

BOYLE, P., MACFARLANE, G.J., MCGINN, R. & 4 others (1990). International epidemiology of head and neck cancer. In de Vries, N. & Gluckman, J.L. (eds.), *Multiple Primary Tumors in the Head and Neck*. pp. 80. G. Thieme Verlag, Stuttgart.

BENDICH, L. & LANGSETH, H. (1989). Safety of vitamin A. *Am. J. Clin. Nutr.*, **49**, 358.

BYERS, T., VENA, J., METTLIN, C., SWANSONS, M. & GRAHAM, S. (1984). Dietary vitamin A and lung cancer risk: an analysis by histologic subtypes. *Am. J. Epidemiol.*, **120**, 7696.

BYERS, T. (1988). Diet and cancer: any progress in the interim? *Cancer*, **62**, 1713.

COLDITZ, G.A., STAMPFER, M.J. & WILLETT, W.C (1987). Diet and lung cancer: a review of the epidemiologic evidence in humans. *Arch. Intern. Med.*, **147**, 157.

COTGREAVE, I.A., GRAFSTROM, R.C. & MOLDEUS, P. (1986). Modulation of pneumotoxicity by cellular glutathione and precursors. *Bull. Eur. Physiol. Pathol. Respir.*, **22** (Suppl), 2635.

DE FLORA, S., BENNICELLI, C., ZANACHI, P. & 4 others (1984). *In vitro* effects of N-acetylcysteine on the mutagenicity of direct acting compounds and procarcinogens. *Carcinogenesis*, **5**, 505.

DE FLORA, S. (1984). Detoxification of genotoxic compounds as a threshold mechanism limiting their carcinogenicity. *Toxicol. Pathol.*, **12**, 337.

DE FLORA, S., BENNICELLI, C., CAMOIRANO, A. & SERRA, D. (1985). *In vivo* effects of N-acetylcysteine on glutathione metabolism and on the biotransformation of carcinogenic and/or mutagenic compounds. *Carcinogenesis*, **6**, 1735.

DE FLORA, S., ASTENGO, M., SERRA, D. & BENICELLI, C. (1986). Prevention of induced lung tumours in mice by dietary N-acetylcysteine. *Cancet Lett.*, **32**, 235.

DE FLORA, S., BENICELLI, C. & CAIMALANO, R. (1989). Inhibition of mutagenesis with N-acetylcysteine (NAC). In. Cerutti, P.A. (ed.), *Anticarcinogenesis and Radiation Protection*, p. 373. Plenum Press: Milano.

DE FLORA, S., D'AGOSTINI, F., IZOTTI, A. & BALAUSKY, R. (1991). Prevention by N-acetylcysteine of benzo(a)pyrene clastogenicity and DNA adducts in rats. *Mutation Res.* (in press).

DE VRIES, N. & SNOW, G.B. (1986). Multiple primary tumours in laryngeal cancer. *J. Laryngol. Otol.*, **100**, 915.

DE VRIES, N., VAN DER WAAL, I. & SNOW, G.B. (1986). Multiple primary tumours in oral cancer. *Int. J. Maxillofac. Surg.*, **15**, 85.

DE VRIES, N., DE LANGE, G., DREXHAGE, H.A. & SNOW, G.B. (1987*a*). Immunoglobulin allotypes in head and neck cancer patients with multiple primary tumors. *Acta Otolaryngol.*, **104**, 187.

DE VRIES, N., DE WAAL, L.P., DE LANGE, G., DREXHAGE, H.A. & SNOW, G.B. (1987*b*). HLA antigens and immunoglobulin allotypes in head and neck cancer patients with and without multiple primary tumors. *Cancer*, **60**, 957.

DE VRIES, N. & SNOW, G.B. (1990). Relationship of vitamins A and E and beta-carotene serum levels to head and neck cancer patients with and without second primary tumors. *Eur. Arch. Orl.*, **247**, 368.

DE VRIES, N., VAN ZANDWIJK, N., PASTORINO, U., MCVIE, J.C., DALESIO, O. & SNOW, G.B. (1990). EUROSCAN. *Euroscan. Eur. Cancer News*, **3**, 1.

DE VRIES, N. (1990). The magnitude of the problem. In de Vries, N. & Gluckman, J.L. (eds), *Multiple Primary Tumors in the Head and Neck*. p. 1, G. Thieme Verlag: Stuttgart.

EUROSCAN STEERING COMMITTEE (1990). Euroscan: EORTC study on screening and chemoprevention with vitamin A and/or N-acetyl-cystein. *Eur. Cancer News*, **3**, 12.

FERRARI, V. (1980). Safety and drug interactions of oral acetylcysteine related to utilization data. *Eur. J. Respir. Dis.*, **61**, 3.

FEX, G., WAHLBERG, P., BIORKLUND, A., WENNERBERG, J. & WILLEN, R. (1986). Studies of cellular retinol-binding protein (CRBP) in squamous-cell carcinomas of the head and neck region. *Int. J. Cancer*, **37**, 217.

FRIEDMAN, G.D., BLANER, W.S. & 6 others (1986). Serum retinol and retinol-binding protein levels do not predict subsequent lung cancer. *Am. J. Epidemiol.*, **123**, 781.

GOEPFERT, H. (1984). Are we making any progress? *Arch. Otolaryngol.*, **11**, 562.

GLUCKMAN, J.L., CRISSMAN, J.D. & DONEGAN, J.O. (1980). Multicentric squamous cell carcinoma of the upper aerodigestive tract. *Head Neck Surg.*, **3**, 90.

GLUCKMAN, J.L. & CRISSMAN, J.D. (1983). Survival rates in 548 patients with multiple neoplasms of the upper aerodigestive tract. *Laryngoscope*, **93**, 71.

HOLOYE, P.Y., DUELGE, J., HANSEN, R.M., RITCH, P.S. & ANDERSON, T. (1983). Prophylaxis of iphosphasmide toxicity with oral acetylcysteine. *Semin. Oncol.*, **100** (Suppl), 66.

HONG, W.K., LIPPMAN, S.M., ITRI, L.M. & 10 others (1990). Prevention of second tumors with isotretinoin in squamous-cell carcinoma of the head and neck. *New. Engl. J. Med.*, **323**, 795.

HORDIJK, G.J. & DE JONG, J.M.A. (1983). Synchronous and metachronous tumours in patients with head and neck cancer. *J. Laryngol. Otol.*, **97**, 619.

LIPPMAN, S.M., LEE, J.S., LOTAN, R., HITTELMAN, W., WARGOVICH, M.J. & HONG, W.K. (1990*a*). Biomarkers as intermediate endpoints. *J. Natl Cancer Inst.*, **82**, 555.

LIPPMAN, S.M. & HONG, W.K. (1989). Second malignant tumors in head and neck squamous cell carcinoma: the overshadowing threat for patients with early stage disease. *Int. J. Radiat. Oncol. Biol. Phys.*, **17**, 691.

MENKES, M.S., COMSTOCK, G.W., VUILLEUMIER, J.P., HELSING, K.J., RUDER, A.A. & BROOKMEYER, R. (1986). Serum betacarotene, vitamins A and E, selenium, and the risk of lung cancer. *N. Engl. J. Med.*, **315**, 1250.

MIDDLETON, B., BYERS, T., MARSHALL, J. & GRAHAM, S. (1986). Dietary vitamin A and cancer. *Nutr. Cancer*, **8**, 107.

PASTORINO, U., SORESI, E., CLERICI, M. & 5 others (1988). Lung cancer chemoprevention with Retinol Palmitate. *Acta Oncologica*, **27**, 1.

PASTORINO, U., CHIESA, G., INFANTE, M. & 5 others (1991*a*). Safety of high dose vitamin A. *Oncology*, **48**, 131.

PASTORINO, U., INFANTE, M., CHIESA, G. & 5 others (1991*b*). Lung cancer chemoprevention. In Pastorino, U. & Hong, W.K. (eds), *Chemoimmuno Prevention of Cancer*, p. 147. G. Thieme Verlag, Stuttgart.

PETO, R., DOLL, R., BUCKLEY, J.D. & SPORN, M.B. (1981). Can dietary beta-carotene materially reduce human cancer rates? *Nature*, **290**, 201.

PRESCOTT, L.F., ILLINGWORTH, R.N., CRICHLEY, J.A.J.H., STEWART, M.J., ADAM, R.H. & PROUDFOOT, X. (1979). Intravenous N-acetylcysteine: the treatment of choice for paracetamol poisoning. *Br. Med. J.*, **11**, 1097.

ROTHMAN, K., CANN, C.I., FLANDERS, D. & FRIED, M.P. (1980). Epidemiology of laryngeal cancer. *Epidemiol. Rev.*, **2**, 196.

SCHANTZ, S.P. & HSU, T.C. (1989). Mutagen-induced chromosome fragility within peripheral blood lymphocytes of head and neck cancer patients. *Head Neck Surg.*, **11**, 337.

SCHANTZ, S.P., SPITZ, M.R. & HSU, T.C. (1990). Mutagen sensitivity in patients with head and neck cancers: a biological marker for risk of multiple primary malignancies. *J. Natl Cancer Inst.*, **82**, 1773.

SCHATZKIN, A., FREEDMAN, L.S., SCHIFFMAN, M.H. & DAWSEY, S.M. (1990). Validation of intermediate endpoints in cancer research. *J. Natl. Cancer Inst.*, **82**, 1747.

SILVERMAN, S., RENSTRUP, G. & PINDBORG, J. (1963). Studies in oral leukoplakias: III. Effects of vitamin A comparing clinical, histopathologica, cytologic and hematologic responses. *Acta Odont. Scand.*, **21**, 271.

SPITZ, M.R., FUEGER, J.J., BEDDINGFIELD, N.A. & 4 others (1989). Chromosome sensitivity to bleomycin-induced mutagenesis, an independent risk factor for upper aerodigestie tract cancers. *Cancer Res.*, **49**, 4626.

STAMPFER, M.J., BURING, J.E., WILLETT, W., ROSNER, B., EBERLEIN, K. & HENNEKENS, C.H. (1989). The 2 × 2 factorial design: its application to a randomized trial of aspirin and carotene in U.S. physicians. *Statistics Med.*, **4**, 111.

TEPPERMAN, B.S. & FITZPATRICK, P.J. (1981). Second respiratory and upper digestive tract cancer after oral cancer. *Lancet*, **2**, 547.

TUYNS, A.J. (1979). Epidemiology of alcohol and cancer. *Cancer Res.*, **39**, 2840.

VAN ZANDWIJK, N. N-acetylcysteine in chemoprevention. In Pastorino, U. & Hong, W.K. (eds), *Chemoimmuno Prevention of Cancer*, p. 210. G. Thieme Verlag, Stuttgart.

VRABEC, D.P. (1979). Multiple primary malignancies of the upper aerodigestive system. *Ann. Otol. Rhinol. Laryngol.*, **88**, 846.

WAGENFELD, D.J.H., HARWOOD, A.R., BYRCE, D.P., VAN NOSTRAND, P. & DE BOER, G. (1981). Second primary respiratory tract malignant neoplasms in supraglottic carcinoma. *Arch. Otolaryngol.*, **102**, 135.

WILLIAMS, R.R. & HORN, J.W. (1977). Association of cancer sites with tobacco and alcohol consumption and socioeconomic status of patients: interview study from the third national cancer survey. *J. Natl Cancer Inst.*, **58**, 252.

WILPART, M., SPEDER, D. & ROBERTFROID, M. (1986). Anti-initiation activity of N-acetylcysteine in experimental colonic carcinogenesis. *Cancer Lett.*, **31**, 319.

WYNDER, E.L., BROSS, I.D.J. & DAY, E. (1956). A study of environmental factors in cancer of the larynx. *Cancer*, **9**, 86.

WYNDER, E.L., BROSS, I.D. & FELDMAN, R.M. (1957). A study of etiological factors in cancer of the mouth. *Cancer*, **10**, 1300.

ZIEGLER, R.G., MASON, T.J., STEMHAGEN, A. & 4 others (1984). Dietary carotene and vitamin A and risk of lung cancer among white men in New Jersey. *J. Natl Cancer Inst.*, **73**, 1429.