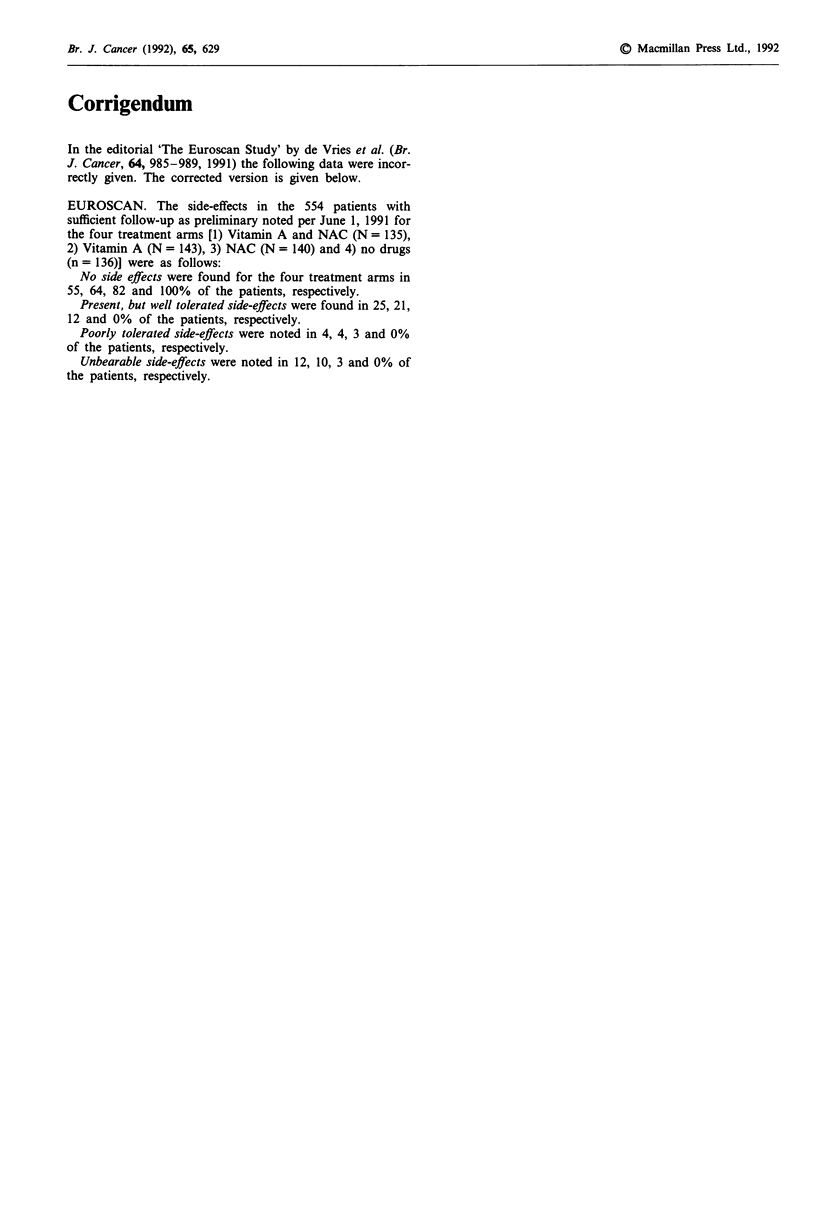# Corrigendum

**Published:** 1992-04

**Authors:** 


*Br. J. Cancer* (1992), **65**, 629 © Macmillan Press Ltd., 1992

### Corrigendum

In the editorial 'The Euroscan Study' by de Vries *et al.* (*Br. J. Cancer*, **64**, 985–989, 1991) the following data were incorrectly given. The corrected version is given below.

EUROSCAN. The side-effects in the 554 patients with sufficient follow-up as preliminary noted per June 1, 1991 for the four treatment arms [1) Vitamin A and NAC (N = 135), 2) Vitamin A (N = 143), 3) NAC (N = 140) and 4) no drugs (n = 136)] were as follows:

*No side effects* were found for the four treatment arms in 55, 64, 82 and 100% of the patients, respectively.

*Present, but well tolerated side-effects* were found in 25, 21, 12 and 0% of the patients, respectively.

*Poorly tolerated side-effects* were noted in 4, 4, 3 and 0% of the patients, respectively.

*Unbearable side-effects* were noted in 12, 10, 3 and 0% of the patients, respectively.